# A Pilot Study to Create a Culture of Innovation and Quality: Focus on a Nursing Association, Credentialing Center, and Foundation

**DOI:** 10.3390/nursrep15090313

**Published:** 2025-08-26

**Authors:** Marcela Cámpoli, Tanya Mulvey, Olivia Lemberger, Hannah Person, Kasey Bellegarde-Armstrong, Oriana Beaudet

**Affiliations:** 1 Institute for Nursing Research and Quality Management, American Nurses Enterprise, 8403 Colesville Road, Suite 500, Silver Spring, MD 20910, USA; tanya.mulvey@ana.org (T.M.); hannah.person@ana.org (H.P.); 2 Innovation Department, American Nurses Enterprise, 8403 Colesville Road, Suite 500, Silver Spring, MD 20910, USA; olivia.lemberger@ana.org (O.L.); oriana.beaudet@ana.org (O.B.); 3 Formerly American Nurses Enterprise, 8403 Colesville Road, Suite 500, Silver Spring, MD 20910, USA; kbellegardearmstrong@hcwh.org

**Keywords:** innovation, quality management, continuous improvement, nursing, healthcare, organizational culture, credentialing, association, assessment, leadership

## Abstract

**Background/Objectives**: In today’s rapidly evolving healthcare landscape, fostering a culture of innovation and continuous improvement is essential—especially within a nursing association that leads individual and organizational credentialing. **Methods**: Colleagues from the American Nurses Enterprise (ANE) Innovation Department and the Institute for Nursing Research and Quality Management collaborated to develop the *Culture of Innovation and Quality Model*^TM^. This process involved conducting a literature review, developing a survey instrument, and administering a pilot pre-survey to ANE employees to collect baseline data. Future research will include a comparison with a post-survey after interventions aimed at strengthening the culture of innovation and quality. **Results**: The results of the pilot pre-survey were high overall and guided the team in identifying areas with the greatest opportunities for improvement. Based on these findings, interventions are being developed that will be implemented at ANE to enhance the practice of and promote the synergy between innovation and quality. **Conclusions**: Achieving and sustaining high-quality standards of care and advancing the professional development of nurses requires a culture where staff feel safe and have opportunities to create, innovate, improve, and learn. This will help promote an environment where people thrive while ensuring that the nursing profession and practice remain cutting-edge and aligned with emerging technologies and evolving healthcare complexities. The *Culture of Innovation and Quality Model*^TM^ may provide a blueprint for organizations who seek to advance innovation and quality knowledge, engagement, and practices and assist their employees in providing better service to colleagues, partners, and customers while adapting to the evolving healthcare environment.

## 1. Introduction

Today’s fast-paced and complex work environment requires an organizational culture that embraces new ideas and strives for excellence. Burgeoning factors such as the coronavirus pandemic, artificial intelligence, including political and social shifts have necessitated new approaches for organizational innovation and experimentation, and created an imperative for capacity building, timely knowledge sharing, and organizational prioritization of research and practice [1]. Healthcare organizations that actively practice innovation through nurturing new ideas, scanning and planning for change, centering people and their needs, and being unafraid to take risks are well-equipped to respond nimbly to complex challenges and navigate uncertainty with strategic, future-focused action. At the same time, organizations that embody a spirit of continuous improvement, implementing processes and systems to meet quality standards and customer expectations, set the bar for standards of excellence in delivering high-quality, cutting-edge products and services.

Increasingly, organizations are recognizing the synergistic relationship of innovation and quality, seeking opportunities to connect these complimentary concepts in high-performing work cultures. The methods of innovation and quality share multiple similarities that when implemented together may provide deeper understanding of customer needs [2] and enhance the impact of national associations. Both innovation and quality approaches embrace a culture of inquiry and learning, prioritize customer/user needs, and employ methods to produce data-driven and novel work. Cultures that integrate innovation and quality create opportunities for idea generation which may drive standards for excellence and provide transparency around the evolving nature of healthcare.

Cultures that support innovation, wellness, and evidence-based practices have demonstrated significant positive correlations with provider well-being, mental and physical health, and job satisfaction [3]. Leadership support has been routinely identified as crucial for the successful implementation and long-term sustainability of innovation and quality endeavors. Research exploring organizational culture and knowledge management in the high-technology industry found that supportive and participative leaders were more likely to increase the efficiency of knowledge management practices leading to enhanced innovation capabilities [4]. Oftentimes innovation and quality initiatives are considered from a top-down (leadership to staff) approach [5]. However, to successfully achieve a culture of innovation and quality, all staff need the knowledge, resources, and support to lead and engage in innovation and quality practices. A thriving innovation and quality culture necessitates a shift in which employees view the tasks that are routinely associated with innovation and quality practices to become second nature and infused into daily work [6].

Nursing associations play a crucial role in addressing health challenges by reimagining healthcare ecosystems, improving access to high-quality care, and promoting the advancement of nursing through research and evidence-based practices. Nurses and nursing allies serve as key advocates, co-designers and co-collaborators in the creation of high-performing healthcare cultures that prioritize teamwork, innovation, and a commitment to quality and excellence in patient care. In 2024, colleagues from the American Nurses Enterprise (ANE) Innovation Department and Institute for Nursing Research and Quality Management came together to collaborate on an initiative to promote and foster an organizational culture of innovation and quality. This initiative directly supports the ANE 2024 strategic plan goal of *enabling operational excellence with an objective to deliver data-driven and customer focused excellence through enhanced and agile technology and innovation*. It also aligns with the successful ISO 9001:2015 [7] quality management certification of the American Nurses Credentialing Center (ANCC), one of ANE’s three core entities. The team designed a study for which a new organizational innovation and quality assessment was developed to support data-driven decisions. The goal of this study is to measure the effectiveness of interventions designed to promote a thriving culture of innovation and quality practices. This initiative is particularly significant, as research on fostering a culture of innovation and quality in the workplace remains limited, with neither a standardized survey nor comprehensive data on the impact of combined innovation and quality initiatives published to date. Building off existing organizational strengths may be an approach to advance innovation and quality synergies. As Nowak [8] stated, “quality and innovation processes are interlinked and should not be treated separately.” Studies have shown that innovation and quality approaches should not be viewed separately, disputing the misunderstanding that to achieve innovation, quality must be discarded. Integrated approaches researched by Zeng and colleagues [9] found that quality management provided a foundation for innovation to occur.

When collaborating across organizations, it is imperative to incorporate shared definitions to ensure a mutual understanding of the application and incorporation of concepts. One of the initial steps of the collaborative journey was to examine multiple definitions of innovation and quality. This process required multiple meetings with protected time for in-depth conversations on the meanings, overlap, and shared understanding of how these definitions could be used synergistically. This study used Lachman and colleagues’ [10] definition of *innovation*: “the application of creativity or problem solving that results in a widely adopted strategy, product, or service that meets a need in a new and different way” and the ISO 9001:2015 [7] definition of *quality:* “The totality of features and characteristics of a product or service that bear on its ability to satisfy stated or implied needs.” Further study is needed to identify the overlapping areas that highlight the desired balance between quality standardization and the need for novel innovative approaches.

**Figure 1:**
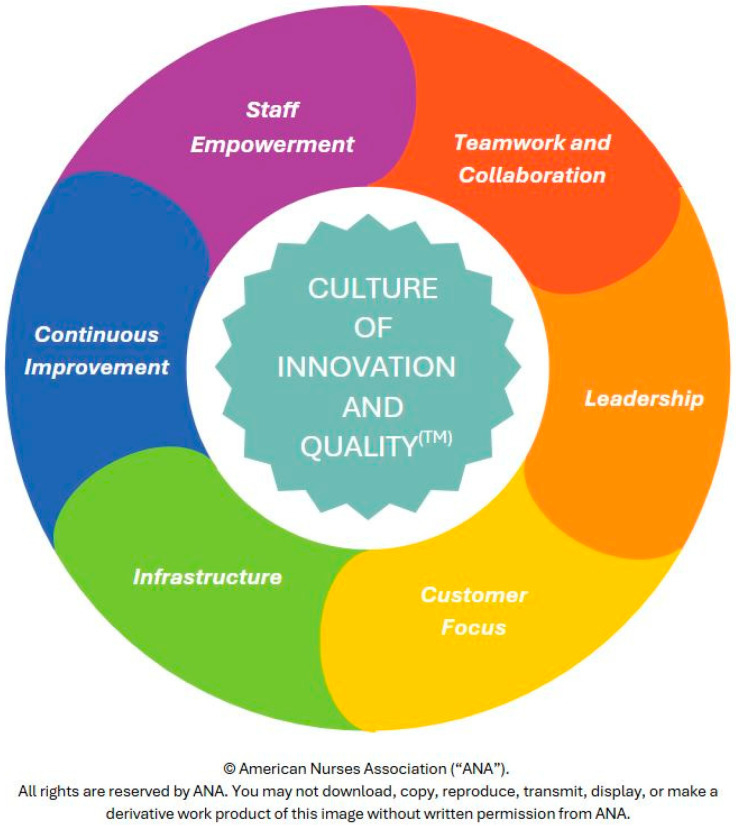
*Culture of Innovation and Quality Model*^TM^.

The *Culture of Innovation and Quality Model*^TM^ was adapted based on the National Association of County and City Health Officials (NACCHO) Version 2.0 2018 Roadmap to a Culture of Quality [6]. The concept in [Figure 1] represents the synergistic components of innovation and quality that were used in designing the pilot pre-survey questionnaire. The pilot pre-survey highlighted six key domains that collectively foster a culture of innovation and quality: staff empowerment, teamwork and collaboration, leadership, customer focus, infrastructure, and continuous improvement. Synergistic methods to support a thriving culture of innovation and quality practices include finding ways to use new tools and strategies; creative problem solving to meet customer needs; developing, redesigning, or implementing work processes; collaborative brainstorming with colleagues; and looking for ways to improve operational efficiency, effectiveness, and quality of work.

## 2. Materials and Methods

This descriptive, pre-post pilot study on the culture of innovation and quality at a non-profit nursing association surveyed a population of full- and part-time employees of the ANE. The study received exempt determination from an IRB. The objectives of the study were to quantitively summarize: (1) pre-intervention baseline attitudes toward innovation and quality knowledge, engagement, and practices; (2) post-intervention measures of employee attitudes toward innovation and quality knowledge, engagement and practices after one year of innovation and quality education and guidance compared to baseline levels of knowledge, engagement and practices; and (3) employee perceptions of changes in innovation and quality knowledge, engagement, and practices during the year. This article is only focused on the study design and administration of the pilot pre-survey related to objective one because the study is still ongoing.

The research team was formed from a collaboration between the ANE Innovation Department and the Institute for Nursing Research and Quality Management. First, a literature review was conducted on the intersections between innovation and quality to develop background information and identify gaps in the literature. Additionally, these sources were used to inform definitions for innovation and quality, and to find synergistic frameworks and theories bridging these concepts. Because no framework or survey instrument was found that combined the assessment of these two concepts, the research team developed the *Culture of Innovation and Quality Model*^TM^, which was adapted from NACCHO’s Roadmap to a Culture of Quality [6], and then created a new instrument based on this model.

The research team designed the survey instrument to have two questions to assess each of the six components of the model, repeated separately for innovation and quality. Additional questions were included to measure perceptions of overall level of innovation and quality within the organization. Feedback on question wording and face validity was gathered from the internal ANE Scholarly Collaborative workgroup. After further revisions, the instrument was pilot tested by an internal group of volunteer employees from various departments. Further revisions were made to the instrument based on pre-survey pilot testing and internal stakeholder feedback. The survey introduction explained the scope of the study and stated the purpose of the initiative was “to support and promote a thriving culture of innovation and quality practices” at the organization. Participants were informed that the survey was anonymous and results would only be reported in aggregate. Consent to participate and confirmation that respondents were 21 years old or older was obtained via a survey question. Upon providing consent, participants were given definitions of innovation and quality with examples of activities related to innovation and quality. This was done to ensure understanding of these concepts and to enhance response validity. The initial questions asked employees to rate their level of knowledge and engagement in activities related to innovation and quality over the past year. Next, employees indicated their level of agreement with two innovation and two quality statements for each of the six components of the *Culture of Innovation and Quality Model*^TM^: staff empowerment, teamwork and collaboration, leadership, customer focus, infrastructure, and continuous improvement. Then employees rated their perception of the current level of innovation and quality at the organization and indicated how familiar they were with innovation and quality resources provided by the organization. The survey concluded with an open-ended question to collect feedback or suggestions on implementing innovation and quality across the organization. Demographics were collected on entity, tenure with the organization, and management versus non-management position to examine representation of respondents and to compare differences between these groups. The ANE includes three distinct entities: a professional association, a credentialing center, and a foundation. The credentialing center is ISO 9001:2015 certified for quality management. For an overview of the complete survey outline, see Appendix A.

The researchers used various survey design methods in an effort to improve data quality and reduce potential bias. To prevent order effects, the six components were presented to participants in random order; however, the innovation statement always preceded the quality question to avoid respondent confusion. Separate items were used to measure innovation and quality so participants could respond about one concept at a time and to measure innovation and quality independently. The 4-point scale ranged from strongly disagree to strongly agree. A midpoint option was not included due to the difficulty of interpretation for both respondents and researchers and to strongly encourage respondents to provide evaluative responses based on their own experiences and perceptions wherever possible. The scale also included an unsure option with a required open-ended text field to understand why respondents may have difficulty rating particular statements. The unsure option was visually offset to separate it from the 4-point scale.

The survey administration was planned carefully to ensure leadership buy-in and promotion, awareness from all staff, and to coordinate with other internal survey efforts. One week prior to fielding the survey, a pre-notification message was included in the weekly newsletter emailed to all ANE employees as well as posted to the organization’s intranet homepage. Personalized survey invitations were emailed to employees through the organization’s survey platform, Alchemer Survey (July 2024, Alchemer, Louisville, CO, USA). The survey remained open for two weeks with up to two reminder emails sent to those who had not yet completed the survey.

Analysis of the pilot pre-survey included frequencies for employment demographics and each survey question, crosstabs reporting results for each question by management/non-management and entity using Chi-square to test for statistically significant differences in agreement vs. disagreement at a level of *p* ≤ 0.10. Additionally, *t*-tests were conducted for the knowledge and frequency of engagement in activities questions, comparing management status and entity. For all analyses, unsure responses were excluded. Qualitative responses underwent a thematic analysis to report key findings.

## 3. Results

The survey results have two main purposes: first to collect baseline and post-intervention data on employee knowledge, engagement, and practices related to innovation and quality that can be used to measure the effectiveness and impact of interventions one year later, and second, to inform the interventions based on gaps and qualitative feedback identified in baseline data. The results section will only discuss key pilot pre-survey findings for each of the six components of the *Culture of Innovation and Quality Model*^TM^, as well as describe planned interventions.

The pilot pre-survey was sent to all 324 regular full- and part-time ANE employees and received 131 valid responses for a 40% response rate. Responses were considered valid if they answered any of the non-demographic questions. The responses to the pre-survey were 40% from credentialing center employees, 57% from association employees, and 4% from foundation employees. Thirty-seven percent of respondents were in a management position. The tenure of employment with ANE of respondents was: 26% up to 2 years, 27% 2 to 5 years, 47% more than 5 years. In examining demographic results, respondents were representative of employees for entity of employment and management/non-management position. For tenure, those who had been with ANE for more than 5 years were overrepresented (47% vs. 36%).

Qualitative analysis was conducted for narrative responses, which included 21 responses to the question collecting feedback or suggestions on implementing innovation and quality across the organization, 13 comments on the questions about resources, and 42 responses to the open-ended unsure options. Five broad themes were identified from these responses. Themes included Data Integration, Learning & Education, Culture, Change Adoption, and Leadership. The identified themes and responses provided deep insight and informed the planned study interventions and dissemination strategies (see Table 1).

**Figure 2:**
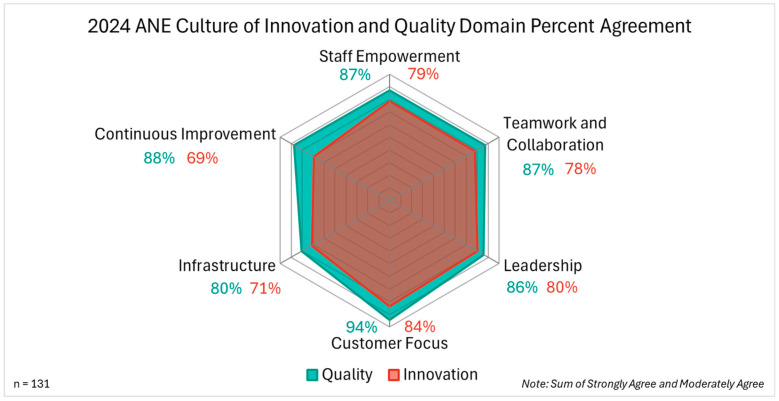
Pilot pre-survey domain composite percent agreement scores.

The survey highlighted six key domains that collectively foster a culture of innovation and quality. Figure 2 displays the innovation and quality composite percent agreement scores for each domain. Employees reported strong results across all areas, with customer focus rated highest and infrastructure identified as the greatest opportunity for growth. While closely aligned, quality consistently outperformed innovation and respondents from the entity that routinely engaged with ISO 9001:2015 quality management standards had statistically significantly higher perception ratings of quality compared to the other areas of the organization (overall quality rating question: *t*(111) = −3.636, *p* < 0.001; domain-specific quality questions: Chi-square *p* < 0.10 for all but one quality question). Additionally, management respondents indicated higher knowledge (innovation: 2.45 vs. 2.11, quality: 2.56 vs. 2.29) and frequency of engagement (innovation: 2.67 vs. 1.63, quality: 3.19 vs. 2.31) in innovation (knowledge: *t*(111) = 3.385, *p* = 0.001; frequency: *t*(111) = 5.194, *p* < 0.001) and quality (knowledge: *t*(109) = 2.423, *p* = 0.017; frequency: *t*(111) = 3.681, *p* < 0.001) than non-management respondents.

### 3.1. Staff Empowerment

Respondents reported high levels of engagement and opportunities to engage in both innovation (79%) and quality (87%). Self-reported engagement opportunities for quality were higher for management (95%) compared with non-management (82%) employees (*X*^2^ (1, *N* = 113) = 4.207, *p* = 0.04). Interventions will include hybrid “Sip, Learn, and Grow” sessions open to all employees to share key survey findings, provide education on available resources, and to engage employees in discussions on how they are implementing innovation and quality in their work, challenges and barriers, and sharing ideas to further promote the culture across the organization.

### 3.2. Teamwork and Collaboration

Respondents similarly reported high levels of teamwork and collaboration for innovation (78%) and quality (87%). Survey results found that respondents reported higher levels of collaboration within departments than across multiple departments. Despite these findings, the organization has many examples of cross-department collaboration, including committees, workgroups, meetings, events, initiatives, and projects. This suggests that the opportunities to work across teams may be unbalanced and dependent upon factors such as department, level, and role. Another factor that may be impacting teamwork and collaboration is that nearly all employees have been working remotely while the headquarters was being moved to a new location. It will be interesting to see how the new office space, designed to promote collaboration, may impact these opportunities. Interventions related to teamwork and collaboration will include education on the benefits of working together and sharing strategies to create opportunities to collaborate on innovation and quality to advance organizational strategic goals, improve efficiency, effectiveness and have greater impact on advancing our vision “A healthy world through the power of nursing.”

### 3.3. Leadership

The research team had support from executive leadership to lead this initiative and study with the goal of promoting a thriving culture of innovation and quality across the organization. To demonstrate this support to all employees, survey communications were sent from a chief officer. In the survey results, most respondents felt they had management support to engage in innovation and quality activities. Composite scores of leadership support were high for innovation (80%) and quality (86%). At the same time, qualitative results included suggestions to ensure leadership support for employees to feel empowered to participate in both innovation and quality activities. Interventions will include presenting results and recommendations to leadership, so that they are aware of opportunities for improvement. After this survey was given, organization leadership provided multiple educational opportunities around broader examples of innovation, which all received very high attendance from employees.

### 3.4. Customer Focus

Customer focus was the strongest area for the organization, with the highest results for both innovation (84%) and quality (94%). This shows that the organization has a very strong culture in valuing and meeting customer needs. Interventions will include education about how quality management contributes to customer satisfaction and sharing tools and resources for implementing quality based on customer requirements. Examples include collecting customer feedback, tracking trends, and considering customer requirements in the process of innovation and continuous improvement.

### 3.5. Infrastructure

Results found that most respondents had self-created or drafted performance goals that included activities related to innovation and quality. Somewhat fewer respondents agreed that the organization dedicated resources to support engagement in innovation and quality activities. Overall, 71% of respondents agreed with these statements for innovation and 80% for quality. Additionally, employees were more familiar with resources for quality (58%) compared with innovation (43%); however, there was variation in the level of awareness with specific types of resources for both innovation and quality. Therefore, interventions around infrastructure will focus mostly on increased education, promotion, and communication about resources. Opportunities to provide additional resources will also be explored in discussions with employees in the “Sip, Learn, and Grow” sessions. These planned intervention sessions will be developed based on qualitative survey feedback that requested educational offerings to be scheduled to avoid meal times (e.g., lunch and learns). While resources are currently available on organizational intranet and external webpages, the research team will work to improve the organization, cross-promotion, and awareness of these resources.

### 3.6. Continuous Improvement

While results for continuous improvement were strong in the area of quality (88%), the greatest opportunity for growth was identified in innovation (69%). This included following documented work processes and adopting lessons learned into work processes. Survey questions in this domain received the highest numbers of unsure responses, with qualitative responses indicating a lack of awareness of documented work processes. Interventions will include education on strategies and tools for documenting work processes and adopting lessons learned. While many resources are currently available for both innovation and quality as it relates to continuous improvement, greater awareness and implementation is needed.

## 4. Discussion

The pilot pre-survey served as an initial assessment to guide understanding of the strengths and opportunities for improvement of ANE employees related to innovation and quality based on the *Culture of Innovation and Quality Model*^TM^. Cultures of innovation and quality may differ across an organization. Not surprisingly, our findings indicated that respondents from the entity that routinely engages with ISO 9001:2015 quality management standards had statistically significantly higher perception ratings for quality compared to the other areas of the organization. This helps to inform effective and efficient use of interventions to make a larger impact toward the goal of promoting quality across the entire organization. Pilot study findings revealed that there was more collaboration in innovation and quality activities within departments than between departments. This indicates that intentional focus on innovation and quality practices that occur across departments will be necessary to improve cross-department collaboration and knowledge sharing to facilitate the breakdown of siloed approaches. This approach may aid in creating a shift in which innovation and quality practices become more routine and infused into employees’ daily work practices.

Pilot pre-survey results showed less familiarity of employees with innovation compared to quality. The majority of employees were unfamiliar with innovation resources, work processes, and formal procedures despite reporting having opportunities to engage in innovation. The American Nurses Association Innovation Accelerator Study revealed the importance of fostering and maintaining a learning community for the further development of innovative knowledge [11]. Innovation and quality development require safe learning environments where employees can share ideas, ask questions, acknowledge mistakes, and reflect on their development journey. Increasing awareness of existing resources and providing more opportunities for learning engagement may address the barrier of visibility and familiarity and provide needed transparency on ways to actively translate innovation and quality knowledge into daily activities.

A valuable lesson learned from this initiative is that innovation and quality initiatives may be improved with the use of shared models and tools such as the RASCI model (responsible, accountable, support, consulted, informed). The research team used the RASCI model as a project management tool to clarify the roles and responsibilities throughout this endeavor. This model was pivotal in aiding task completion, shared decision making, time management, and the successful completion of deliverables. For the successful advancement of collaborative initiatives across organizations, increased communication around the use of shared models and tools is essential.

Limitations to this study include that the post-survey portion of this study has not yet been completed. This manuscript reflects a high-level overview of pilot pre-survey findings that will inform the interventions. The high-level findings reflect the need to protect the confidentiality of organizational data. Further research is also needed to validate the survey instrument.

Next steps of this study include interventions related to pilot pre-survey findings. An infographic was created to share pilot pre-survey results, highlight opportunities for growth, and provide internal links to innovation and quality resources. The conceptual *Culture of Innovation and Quality Model*^TM^ will be used to guide our interventions for the various domains including staff empowerment and infrastructure (Sip Learn and Grow sessions), teamwork and collaboration (educational offerings), leadership (pilot pre-survey results presentation), customer focus and continuous improvement (sharing innovation and quality tools, office hours). Intervention strategies will need to move from an understanding of the facts of innovation and quality to a deeper understanding of how innovation and quality practices when employed synergistically can lead to a thriving culture, achieving more than one approach could accomplish independently. Dissemination opportunities such as presentations and webinars will be conducted both nationally and internationally. A post-intervention survey will be used to measure ANE employees’ attitudes toward, knowledge of, and engagement in innovation and quality, as well as familiarity with innovation and quality education and guidance compared to the initial level of innovation and quality knowledge, engagement, and practices. The research team intends to publish additional articles and share further findings on the effectiveness of the interventions after the completion of the post-intervention survey.

## 5. Conclusions

A central tenet of the journey as co-collaborators at the ANE has focused on the exploration and amplification of innovation and quality synergies that can be used to galvanize a thriving organizational culture. Sustainability efforts to promote standards of care and professional development require a culture of innovation and quality where staff feel safe and have opportunities to create, collaborate, and learn. The *Culture of Innovation and Quality Model*^TM^ may provide a blueprint for organizations who seek to advance innovation and quality knowledge, engagement, and practices and assist their employees in providing better service to colleagues, partners, and customers while adapting to the evolving healthcare environment. Continued emphasis on the synergistic and complementary approaches to innovation and quality practices requires collective support and further research to propel healthcare environments towards a flourishing future. These synergies may illuminate the underlying factors that make meaningful engagement in innovation and quality practices a sustained reality.

## Figures and Tables

**Table 1:** Planned Study Interventions and Dissemination.

Presentations to American Nurses Enterprise Leadership, Employees, Scholarly Collaborative
External Presentations (International/National Nursing Conferences, Webinar, ANE Research Advisory Council)
Internal dissemination of Infographic with Summary of Key Findings
Educational Offerings (Sip, Learn, and Grow, Elevating the Enterprise Modules)
Office Hours
Ongoing Communication (Intranet, Newsletter)

## Data Availability

The datasets presented in this article are not readily available because the study is ongoing and the information is proprietary to the American Nurses Enterprise.

## Public Involvement Statement

No public involvement in any aspect of this research.

## Guidelines and Standards Statement

This manuscript was drafted against the Revised Standards for Quality Improvement Reporting Excellence (SQUIRE 2.0).

## Appendix A

Survey questionnaire outline:

1. Introduction
  - Consent question
  - Innovation and quality definitions and examples
2. Knowledge
  - Rating question of knowledge about innovation
  - Rating question of knowledge about quality
3. Frequency
  - Rating question of frequency of engagement in innovation
  - Rating question of frequency of engagement in quality
4. Randomized domain sections:
  - Staff Empowerment
    - Agreement question 1 for innovation
    - Agreement question 1 for quality
    - Agreement question 2 for innovation
    - Agreement question 2 for quality
  - Teamwork and Collaboration
    - Agreement question 1 for innovation
    - Agreement question 1 for quality
    - Agreement question 2 for innovation
    - Agreement question 2 for quality
  - Leadership
    - Agreement question 1 for innovation
    - Agreement question 1 for quality
    - Agreement question 2 for innovation
    - Agreement question 2 for quality
  - Customer Focus
    - Agreement question 1 for innovation
    - Agreement question 1 for quality
    - Agreement question 2 for innovation
    - Agreement question 2 for quality
  - Infrastructure
    - Agreement question 1 for innovation
    - Agreement question 1 for quality
    - Agreement question 2 for innovation
    - Agreement question 2 for quality
  - Continuous Improvement
    - Agreement question 1 for innovation
    - Agreement question 1 for quality
    - Agreement question 2 for innovation
    - Agreement question 2 for quality
5. Overall
  - Rating question for level of innovation
  - Rating question for level of quality
6. Resources
  - Rating question for familiarity of resources for innovation
  - Rating question for familiarity of resources for quality
7. Open-ended feedback or suggestions question
8. Demographics
